# Resilience from the ground up: how are local resilience perceptions and global frameworks aligned?

**DOI:** 10.1111/disa.12342

**Published:** 2019-04-04

**Authors:** Emilie Beauchamp, Jennifer Abdella, Susannah Fisher, John McPeak, Hannah Patnaik, Papa Koulibaly, Daouda Cissé, Mamadou Touré, Aly Bocoum, Momath Ndao, Yacouba Deme, Bara Gueye

**Affiliations:** ^1^ Researcher (Climate and Environment), Strategy and Learning Group, International Institute for Environment and Development United Kingdom; ^2^ Director of Climate Resilient Development, Near East Foundation United States; ^3^ Senior Researcher, Climate Change, International Institute for Environment and Development United Kingdom; ^4^ Professor, Public Administration and International Affairs, Maxwell School of Citizenship and Public Affairs Syracuse University United States; ^5^ PhD student at Public Administration and International Affairs, Maxwell School of Citizenship and Public Affairs Syracuse University United States; ^6^ Head of Monitoring and Evaluation and Knowledge Management, IED (Innovation Environnement Développement) Afrique Senegal; ^7^ Head of Monitoring and Evaluation and Knowledge Management, Near East Foundation Mali; ^8^ DCF Policy Officer, IED (Innovation Environnement Développement) Afrique Senegal; ^9^ Programme Lead, Decentralising Climate Funds, Near East Foundation Mali; ^10^ Programme Lead, Decentralising Climate Funds, IED Afrique Senegal; ^11^ Mali Country Director, Near East Foundation Mali; ^12^ Senegal Country Director, IED Afrique Senegal

**Keywords:** climate adaptation, food security, resilience, Sahel, subjective indicators, well‐being

## Abstract

Numerous resilience measurement frameworks for climate programmes have emerged over the past decade to operationalise the concept and aggregate results within and between programmes. Proxies of resilience, including subjective measures using perception data, have been proposed to measure resilience, but there is limited evidence on their validity and use for policy and practice. This article draws on research on the Decentralising Climate Funds project of the Building Resilience and Adaptation to Climate Extremes and Disasters programme, which supports communities in Mali and Senegal to improve climate resilience through locally controlled adaptation funds. It explores attributes of resilience from this bottom‐up perspective to assess its predictors and alignment with food security, as a proxy of well‐being. We find different patterns when comparing resilience and the well‐being proxy, illustrating that the interplay between the two is still unclear. Results also point to the importance of contextualising resilience, raising implications for aggregating results.

## Introduction

With the increase in climate shocks and rising awareness of the impacts of climate change, the concept of resilience has become increasingly prominent across academia, policy and practice in environmental and social spheres (Eakin et al., 2014). Resilience was originally defined as a quality of an ecological system, describing its ability to absorb disturbance without falling into another state or phase, while maintaining its essential functions (Holling, 1973). The concept has since been adopted and redefined in diverse fields, with various applications and meanings within ecology, engineering, literature, psychology, health, development and climate science (Brown, 2016). Definitions of resilience from a socio‐ecological perspective also cover a system's capacity to self‐organise and adapt to emerging circumstances (Gunderson, 2000; Folke, 2006). Definitions from a primarily social perspective add the ability to learn, innovate and change (Adger et al., 2011).

The original theoretical concept from socio‐ecological systems work defines resilience as a quality that is norm‐free, transferable across disciplines and scale‐independent. It can be linked to both positive and negative outcomes in ecological and socioeconomic domains, reflecting the context and scale of the system. A resilient socioeconomic system may not be fair if its structures are based on conflict and inequality, for example in an enduring dictatorship. Or it may be perceived as desirable to certain social groups only, for example the ruling classes.

While the concept of resilience itself is free from norms, discipline and scale, it is intrinsically a context‐specific notion. It can thus be applied to describe individuals, households, communities, processes, systems, institutions and ecosystems at local, regional, national and international levels. Increasingly, it is applied within the field of international development and climate change at various scales (Brooks et al., 2014; Brown, 2016). The concept of resilience adds dimensions of capacity and agency to development frameworks traditionally based on vulnerability analyses. This means it can shed light not only on beneficiaries’ passive exposure to particular conditions but also on their ability to manage and respond to stresses or shocks (Béné et al., 2015).

The fluidity of the concept is reflected in the multitude of frameworks attempting to support the measurement of its theoretical multidimensionality into practical assessments, yet with no emerging consensus to date on metrics and methods (Levine, 2014; Schipper and Langston, 2015; Sharifi, 2016). Thus, the concept of resilience is now widely used in policy and practice yet its meaning and measurement remain contested. There is risk in defining resilience narrowly—either in reference to only a specific subset of shocks or stresses or without considering diverse perspectives. Failure to consider the multiple drivers of vulnerability can increase the risk of maladaptation or unsuccessful adaptation (Adger et al., 2011), while conceptions of resilience that ignore local priorities can result in missed opportunities to support existing strategies (such as mobility among pastoral populations). For example, short‐term coping strategies by communities (such as intensifying natural resource use) can lead to long‐term maladaptation (such as ecological degradation) that increases vulnerability to climate shocks.

In the framework of global climate programmes, such as the Building Resilience and Adaptation to Climate Extremes and Disasters (BRACED) programme funded by the UK Department for International Development (DFID), the discussion about how best to measure resilience and aggregate results is ongoing. There is growing interest in the use of subjective measures of resilience through perception indicators, building on research on psychological resilience and subjective well‐being (Clare et al., 2017; Jones and Tanner, 2017). This recent shift in resilience research towards subjective indicators recognises both that observable socioeconomic variables are limited in the measurement of less tangible factors that may contribute to individual or household resilience, and that individuals and households are well‐placed to gauge their own capacities to withstand shocks and stresses (Jones and Tanner, 2017). This article contributes to this debate by assessing the use of a perception or ‘subjective’ resilience measure, its relationship to other indicators and its potential utility in global frameworks.

This research draws on monitoring and evaluation (M&E) frameworks applied in the Decentralising Climate Funds (DCF) project—a research action and advocacy project supporting local people in Mali and Senegal to become more resilient to climate change through access to locally controlled adaptation funds. We empirically explore the relationship between perceptions of resilience; observable socioeconomic variables thought to contribute to resilience; and food security as a related well‐being variable commonly used in the Sahelien context. We use this analysis to evaluate the reliability of perceptions of resilience as indicators and the implications for donor reporting and aggregation of adaptation data.

In this article, we analyse and explore how household perceptions of resilience through self‐assessment relate to observable variables, and the implications for using such measures to understand local changes or to fulfil donor reporting frameworks’ call to aggregate results. We ask the following questions: How do attributes of self‐assessed resilience among local households correlate with attributes of well‐being (food security) or other observable variables that seek to measure resilience capacities? What are the implications for the use of perception and proxy indicators locally and what can we learn about the potential for aggregation of climate resilience within global frameworks?

We answer these questions by assessing results from qualitative resilience assessments and a baseline survey from the BRACED DCF project that measures households’ self‐assessed resilience and food security in dryland Sahelian contexts, to compare bottom‐up conceptualisations of resilience against a donor‐defined resilience framework. We seek to empirically test the *ex‐ante* hypothesised relationships between the concept of resilience, as articulated in DFID guidance, and a broad array of other measurable variables that capture different dimensions and perspectives of the concept of resilience.

### Concepts of climate resilience within international development

Donor agencies and international climate funds have designed a range of global frameworks and indicators to standardise their use of the term ‘climate resilience', to measure impact and to aggregate results at a national and international level (Bours et al., 2015). Within these frameworks, resilience is often framed normatively as a context‐specific and quantifiable outcome (such as changes in adaptive coping capacities) that is conceptually linked to improved development and well‐being impactrelated variables, such as food security or poverty reduction (Marshall et al., 2010; Bennett and Dearden, 2014; Constas et al., 2014; Béné et al., 2015). The BRACED programme, for example, implies a direct relationship between resilience and longer‐term well‐being, identifying improved resilience as an outcome and well‐being as an impact within the programme logical framework for reporting (BRACED Knowledge Manager, 2015). Additionally, several programmes such as BRACED often define resilience specifically in reference to climate shocks and stresses rather than in terms of wider global shocks and changes, for example political conflict or economic decline. Yet local communities can rarely easily separate their experience of different shocks and stresses (McPeak et al, 2017).

Like the concept of human well‐being, resilience is complex and multidimensional, often linked to desirable development impacts. Yet, unlike well‐being, subjectivity in conceptualising resilience has not received as much attention. It is now widely accepted that well‐being can be understood in terms of three interacting dimensions: the objective material circumstances of a person; a relational dimension capturing the person's ability to achieve those goals through social networks and interactions; and a subjective evaluation by the person of their goals and the processes they engage in to attain them (Gough and McGregor, 2007). Subjective well‐being assessments have been applied across psychology, development, economics and conservation fields to avoid the top‐down, donor‐defined indicators often used in quantitative evaluations (Diener et al., 1999; Vira and Kontoleon, 2012; McKinnon et al., 2016). Studies examining the subjective well‐being indicators point to their validity and correlation with other objective proxies (Kahneman and Krueger, 2006; Oswald and Wu, 2010).

Since the Paris Agreement in 2015, the international community has committed over $10 billion to support climate adaptation (GCF, 2018). Given this level of resources and attention, proper framing of resilience goals and measures takes on new urgency. Definitions of resilience in global programmes matter because the conceptual frameworks used shape how interventions aimed at improving resilience are operationalised and evaluated. This evidence feeds back to inform stakeholders on what has worked, how and for whom—thus directing the next round of interventions (Béné, 2013). With the focus on resilience as a normative ‘outcome’ of development programming, there is growing interest in its measurement to target interventions, monitor activities and aggregate and evaluate results (Brown, 2016). There is substantial debate on resilience indicators, the potential role of subjective indicators (asking individuals how they perceive their household's resilience in relation to specific shocks, timeframes or contexts) and observable variables such as access to assets, services, livelihood capital, safety nets and early warning systems (Béné, 2013; Brooks et al., 2014; Jones and Tanner, 2017).

Maxwell et al. (2015) highlight the importance of using mixed methods to understand resilience and combining subjective measures with others. Jones and Tanner (2017) argue that ‘there will be areas where objective and subjective assessments differ. Understanding the drivers (and biases) for such disparities could point to different interpretations of resilience on the ground'. A rare empirical analysis is found in the work of Jones et al. (2018), using a nationally representative survey in Tanzania to explore the use of a subjective measure.

Although the use of subjective measures of resilience to understand people's own perceptions of their resilience has gained some traction in the literature, empirical exploration has so far been limited. Subjective measures of resilience potentially offer advantages for resilience programming: if effective, they are a resource‐light way to quickly assess changes in particular populations without a need for a series of complex proxies, and they offer a bottom‐up assessment of change—contributing downward accountability to evaluation frameworks to complement the more traditional upward focus to donors and funders. These advantages are particularly salient in a project like DCF, which seeks to institutionalise and support evaluative assessments within local government institutions that have limited human and financial resources.

Béné (2013) highlights the circular nature of measuring resilience, whereby inductive variables that are chosen to be proxies for resilience are targeted by interventions and show improvement in survey results. As Béné stresses, however, this does not prove the relationship between the chosen proxy and resilience itself, only that the proxy has improved over time. Béné argues that a new framework for measuring resilience needs to take account of the fact that resilience is both objective and subjective (which Béné defines as how people feel about their resilience) and be generic enough to be used in different contexts.

Beyond measuring resilience outcomes of any one intervention, there is increasing focus within global programmes and climate funds on aggregating results from local, context‐specific processes to report cross‐portfolio achievements to donors and political actors. This impetus, which has resulted in a focus on creating aggregable quantitative measures, is part of a wider debate on the utility of universal metrics for adaptation (Roehrer and Kouadio, 2015; Craft and Fisher, 2018). As evidenced by DFID's complex guidance for the main resilience indicator for its BRACED programme (KPI4) (DFID, 2014), describing a nine‐step process to defining and measuring the indicator, this is not an easy process. It remains an open question how meaningful the aggregation of different proxies for resilience across multiple projects and contexts really is.

Yet normative goals embedded in the development and resilience discourse can be highly contested. Moreover, political pressures on donor agencies and climate funds to demonstrate clear and timely results are likely to incentivise measures suitable for upwards accountability and aggregation, rather than more nuanced measures that capture local changes in resilience and that could be more useful to local stakeholders. Given the potential for unintended outcomes or mismatched priorities, there is a clear need to consider how local conceptualisations of resilience might be joined up with the resilience frameworks currently in play among international donor communities (Clare et al., 2017).

### The importance of food security

The DCF project operates in a region marked by environmental variability and food insecurity. For smallholder and subsistence farmers in variable environments, climate impacts are often first felt through production losses that affect household food security and nutrition through direct losses of food stocks/harvests or reduced income (FAO, 2013). Thus, vulnerability in this context often translates to food stress. Food security is defined in terms of availability, access and utilisation; a fourth dimension, stability, takes into consideration changes in the first three dimensions over time (FAO, 2008). Climate shocks and stresses most immediately affect the related concepts of food availability and food access, where availability refers to the supply of food from production and exchange and access reflects the economic and physical ability of households to obtain food from all sources, including their own production, stocks, purchases, gathering or food transfers (Bilinsky and Swindale, 2010). Households with greater resilience demonstrate greater food security, as measured using the Household Food Insecurity Access Score (HFIAS) (Béné et al., 2015).

We note that food security is a measure of well‐being; it is not itself a measure of resilience. The relevant consideration, therefore, is not food security at a moment in time, but change (or lack thereof) in food security in the face of shocks and stresses. Over time, the extent to which food production systems are able to reliably cover household food needs in the face of shocks and stresses might therefore be viewed as a measure of the success of adaptive strategies.

## Context

This article focuses on the DCF project, one of fifteen projects of the DFID‐funded BRACED programme. The DCF project aims to support locally led adaptation to climate change in Mali and Senegal, seeking to build resilience by enabling communities to access funding for locally prioritised public good investments (NEF/IIED/IED Afrique Consortium, 2014). The objective is for these public good investments to enhance individual, household and community resilience in the face of climate change.

The DCF mechanism consists of four dimensions: (1) participatory resilience assessments, through which communities identify climate stresses, opportunities and resilience‐building priorities: (2) local climate adaptation funds under discretionary management of local governments and used to finance locally prioritised public good investments in resilience; (3) local adaptation committees that identify and implement resilience investments based on inclusive community consultations and predefined fund criteria; and (4) local monitoring to assess effectiveness of resilience investments, support iterative learning and inform future planning (Hesse, 2017). The project design reflects the principle that local communities have strategies for managing variability and are best placed to identify investments that will support local adaptive strategies. Communities implement locally prioritised adaptive strategies that reduce their vulnerability to climate stresses and shocks. In practice, most local investments aim to reinforce livelihood systems and productive assets.

**Figure 1:**
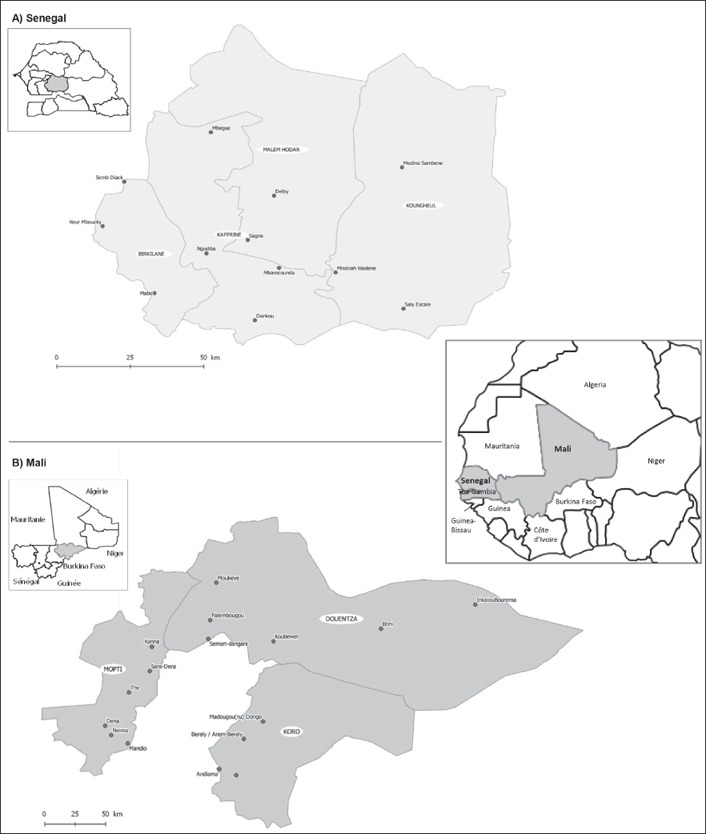
Map of the surveyed villages in Kaffrine, Senegal (A), and in Mopti, Mali (B) **Source**: authors.

The DCF project operates in the region of Kaffrine in Senegal and the region of Mopti in Mali (Figure 1). Both Kaffrine and Mopti comprise diverse agro‐ecological systems and communities with varying access to transportation corridors and markets.

Kaffrine is divided administratively into four *départements* aligning west to east, with three agro‐ecological zones spread north to south: the northern zone bordering the Ferlo agro‐pastoral region, the central peanut basin and the southern more humid zone bordering The Gambia. Running through Kaffrine from west to east are the main railway line and a major international highway. Mali's Mopti region, situated in the Inner Niger River Delta, contains diverse agro‐ecological systems, including flood plain cultivation, nomadic grazing, settled rainfed cultivation, and important fishery resources. A major international route and the Niger river run through this region. Mopti is divided into eight *cercles*, of which DCF works in three: Koro, Mopti and Douentza. Most project beneficiaries across countries engage in agro‐pastoral livelihoods underpinned by natural resources and rainfed agriculture systems that are sensitive to climate change. Most engage in subsistence agro‐pastoral activities; communities experience annual lean or ‘hunger’ seasons at the end of the dry season and start of the rainy season.

In each country, selected communities use a variety of livelihood strategies for household production. Most households identify first as cultivators, growing crops such as millet, groundnuts, cow peas, maize and rice. There are also significant numbers of households that identify predominantly as livestock producers, often utilising seasonal migration to balance livestock feed and water needs with changing seasonal conditions. The presence of riverine systems in both countries supports a small share of the population who identify as fishers. Finally, there are a variety of complementing livelihood occupations such as commerce, artisanal work, the religious leadership and the civil service. Households identified their predominant occupation as one of these activities but very few reported just one type of productive activity in their livelihood strategy; most pursue a portfolio of livelihood activities.

Both Mopti and Kaffrine are vulnerable to slow‐onset climate change (increasing temperatures, desertification, changing rainfall and seasonal riverine flooding patterns) and climate shocks (droughts, flooding, wildfires) that undermine productive systems, livelihoods, food security and well‐being (NEF/IIED/IED Afrique Consortium, 2014). Among other impacts, climate shocks and stresses diminish crop and livestock productivity and undermine rural livelihoods, while repeated exposure to recurrent crises erodes household and community coping capacities (such as stored food or seedstocks) (USAID, 2017). Climate shocks and extremes in the Sahel thus heavily affect both food availability and accessibility (direct production or ability to purchase) and contribute to both chronic and acute food insecurity (Giannini et al., 2017). For example, in 2012, severe drought caused severe food shortages, with over 18 million food‐insecure people in the Sahel (OCHA, 2013).

## Methods

### Research design

Project data collection was based on the DFID guidelines defined in advance of the BRACED programme to operationalise resilience measures for project M&E across the 15 projects. BRACED defines resilience to climate shocks and stresses, which may be intensifying because of climate change, as:

*… a composite attribute possessed by each individual, that represents their ability to anticipate, avoid, plan for, cope with, recover from and adapt to (climate related) shocks and stresses. Improved resilience means that an individual is better able to maintain or improve their well‐being despite being exposed to shocks and stresses* (DFID, 2014).

The programme provides guidelines on operationalising the concept of resilience for M&E—namely through the document Key Performance Indicator 4 (KPI4) (DFID, 2014). KPI4 establishes a framework of measurable variables that touch on different dimensions of resilience at the individual or household level; it is meant to be an aggregable measure across DFID's International Climate Fund projects. Also central to the BRACED effort is a qualitative measure of resilience, the 3As: the ability to adapt to, anticipate and absorb shocks from climate extremes and disasters (Bahadur et al., 2015). As part of an action research programme, our methodology has been partially guided by our engagement with stakeholders and how they may use project data and results in practice. Our aim has been to test indicators that could be used within local planning systems and so we have sought to assess the validity of resource‐light measures of resilience that are ‘grounded in, and applicable to, the local experience’ (Lebel and McLean, 2018). Relevant tools and methods included participatory resilience assessments, household surveys and community investmentlevel theories of change.

### Participatory resilience assessments

The DCF project used a resilience assessment methodology (Keita and Koulibaly, 2017) to develop participatory understandings of local resilience and local theories of change on how resilience could be built. Nine participatory resilience assessments were conducted during workshops attended by representative local actors from representative agro‐ecological zones:^2^ six in the Mopti region of Mali and three in the Kaffrine region of Senegal. In Mali, these workshops were supplemented by interviews with households in six villages in three different agro‐ecological zones.^3^ The assessments began with an analysis of livelihood systems and well‐being; explored different aspects of resilience and what different ‘levels’ of resilience and well‐being look like on a Likert scale; identified the resilience features of different livelihood groups and agro‐ecological zones; and looked at possible investments to improve resilience and well‐being.

The resilience assessments underscored the breadth of the concept of well‐being according to stakeholders, seen by participants as the optimal physical, moral, cultural, social and spiritual state required for a decent life. Terms for well‐being in local languages were discussed and agreed upon (Fulfulde as *neema*; Bambara as *lafia, wassalen, hèrè*; Wolof as *ndeuguerlay*). Although well‐being is often associated with economic goods, the criteria local stakeholders suggested to describe it show a more nuanced understanding that focuses on environmental and especially social aspects as well as economic dimensions. Food security was identified as an important factor of well‐being linked to resilience.

For example, herders from the inner delta area in Mopti region suggested that investments in livestock routes and local conventions would ensure their secure access to aquatic grasslands that are flooded in the rainy season, ensuring their herds’ productivity during the dry season and droughts, and thereby improving their food security and household purchasing power, and ultimately well‐being. Social factors included being married, socially respected, in good health and educated, with peace of mind, good relations with neighbours and social stability; economic factors included food security and good purchasing power; environmental factors included good housing and a healthy environment.

Other criteria for well‐being varied according to sex and age. For example, in Mali, women placed great emphasis on not being subject to sexual violence or sexually transmitted diseases, having a harmonious marriage and being able to pay for their daughters’ wedding. Young fishermen, meanwhile, listed a large motorbike and sound system as important factors in their well‐being. Local stakeholders defined resilience to climate hazards in terms of tenacity or hard work.

The perception among participants was that their communities had low levels of resilience to climate variability and extremes. Stakeholders explained these perceptions through the constraints to their adaptation strategies, for example a lack of financial resources to invest in assets and new livelihoods; difficulty in obtaining quality agricultural inputs and reliable and timely market information; lack of capacity of technical services to maintain natural resource governance institutions; and the presence of conflicts in local natural resource management. The resilience assessments identified that the impact of these constraints on local people—and thus on their level of resilience—varied according to a number of factors, such as level of household and individual well‐being; opportunities and constraints in different agro‐ecological areas; whether adoption of adaptive strategies required significant resources or specialist skills; proximity to large towns; and existence of road networks and reliable telecommunications. These factors form the basis for the selection of the variables used in the quantitative analysis.

### Household survey

In the frame of its programmatic M&E, the DCF team developed a survey instrument to measure resilience and identify changes over time in household resilience guided by the core KPI4 elements (DFID, 2014) and the resilience factors and potential investments identified in the participatory resilience assessments. This article is based on the baseline data collected for a longitudinal analysis to assess resilience outcomes associated with the DCF project. Given this programmatic M&E purpose and the desire to test simple indicators that local actors could use, the DCF team framed resilience in the context of climatic shocks and tested indicators of subjective resilience and food security.

Household survey data were collected in October and November 2015 across 12 villages in Senegal and 16 villages in Mali, with project survey design supported by the DCF project team and Syracuse University. Target villages were selected based on the representativeness of their community characteristics (agro‐ecological zone in Senegal, access to the Niger River in Mali). We further stratified by village population size and distance to markets. Based on the population, we developed a sampling size and interval, specifying 17 households per community for 204 households in Senegal and 25 households per community for 400 households in Mali. The objective of a total sample size of 604 households was selected by conducting Minimal Detectible Effect calculations for household‐level data gathered for another project in the Senegal River Valley. The approximate 2:1 ratio for Mali: Senegal was based on the difference in the population size between the Mopti and Kaffrine regions according to the most recent census data.

Independent enumerators collected survey data after receiving training in the conceptual framework, project activities and questionnaire. Following a pilot, the questionnaire was administered to heads of households randomly selected from a household roster (generally the tax list) obtained in the community from local leaders in each village. A condensed version of the survey was then administered to the household head's spouse, if the household was not female‐headed and a spouse was present. Prior and informed consent was acquired for each survey. Before each interview, the purpose of the research and content of the interview were explained. Participants were told that they were not obliged to participate, that they could stop the interview at any point and that all their answers would be kept confidential. Because of low levels of literacy, participants gave verbal consent.

### Variables and statistical analysis of the survey

Based on the literature and the participatory assessments, we were interested to see how the self‐assessed resilience indicator relates to the food security indicator. The household survey asked each household respondent about their primary livelihood activities and strategies over the past year, along with questions related to their access to resources, infrastructure, markets and services. It then asked how many months of food security they were able to achieve per year as a result of those activities (with possible responses of one to twelve months). This question seeks to understand the household's food access, and it is similar to the US Agency for International Development (USAID)‐developed *Months of Adequate Household Food Provisioning* indicator (Bilinsky and Swindale, 2010). By capturing a household's ability to meet food needs over a year, it can reflect its well‐being linked to variables such as crop production, income, storage, labour availability and occurrence of social or natural disasters. In this context, resilience‐building strategies seek to reduce household vulnerability to specific factors that result in inadequate food provisioning in the face of shocks, ultimately improving the household's well‐being. The indicator of household food provisioning can, over time, capture changes in a household's ability to avoid, adapt to and respond to shocks and stresses to ensure food access.

While there are many indicators of household food security—some more widely adopted by global food programmes—these often require asking respondents a longer series of questions that are then indexed, or the measure is time limited (asking respondents to recall food availability or access over, such as a 24‐hour or 30‐day period). As such, the alternative measures are less light touch and/or do not capture seasonal changes in food security. For example, the HFIAS measure is a multi‐question indexed score that considers food access and perceptions over a four‐week recall period (Béné et al., 2015).

We selected the household provisioning question because it (1) captures a longer (annual) look‐back period, meaning it can reflect seasonal variation and lean periods; (2) captures food access from a number of sources (own production, stocks, purchased, etc.); (3) if measured over time, has the potential to detect shocks and stresses that affect food systems (Lebel and McLean, 2018); (4) is a light‐touch question that local actors could easily adopt; (5) is a measure that, in the DCF team experience, is culturally appropriate—for instance, rural people in Mali and Senegal both understand and know it with respect to their own household economies; and (6) though simple, is well‐correlated with actual household nutritional outcomes (Makamto Sobgui et al., 2018). Other coping capacities can mitigate the impacts of poor food access on household resilience (such as livelihood diversification, migration, remittances, harvesting wild foods), and other survey questions were directed at understanding these. As a commonly understood variable, the food coverage measure offers an additional point of comparison that can be used to triangulate resilience and changes in well‐being over time.

We also directly asked household heads to conduct a self‐assessment of their household's resilience. We asked households, using the local expressions for resilience defined in the scoping phase, to assess how they perceived their household's resilience over the past year on a Likert scale from very weak (1) to very strong (5). Given the low number of observations in the ‘strong’ category, we combined 4 and 5 together for any further analysis, creating a four‐level ordinal variable that can be thought of as very weak (1), weak (2), neither weak nor strong (3), and strong and very strong combined (4).

The resilience self‐assessment was asked following the food security items but prior to other socio‐demographic and contextual questions, to avoid reflection bias in resilience scores. We then asked about the livelihood strategies used over the past year when facing climate variability and shocks, along with which shocks had been experienced, such as bushfires, violent winds, pest and flooding.

As part of the analysis of the livelihood strategy data, we have developed a simple measure of livelihood strategy diversity by counting the number of different categories of activity (cultivation, herding, fishing, commerce, artisanal, etc.) identified. In addition to variables hypothesised to contribute to changes in resilience at the household level, we used community‐level variables to capture community‐specific effects together with the household‐level information. We also allowed for community‐specific impacts on variance, as the error terms are clustered by study site in the regressions.

The conceptual design and choice of simple indicators used in this study mirror the practical realities shaping the implementation of a large multi‐year M&E system. While we recognise a tension between the conceptual literature of resilience and our M&E research design, we support that simple local metrics that are ‘robust enough’ for local decisions are essential for successful programme management, navigating the conceptual space between local utility and global demands.

We ran regressions on both response variables—resilience and food security. An ordinal regression was used to identify the impact of variation in a set of measures on the household's resilience self‐assessment, and negative binomial regressions were used for food security as over‐dispersed count data. For each regression, we conducted the analysis on the country‐specific data and the pooled data for the entire sample for each variable. Based on Chow test results, we were not able to accept the hypothesis that the parameters were the same across both countries. We therefore present country‐specific results for the parameter estimates. For the selection of independent variables, we used a combination of theoretical relationships between variables and the concepts of resilience and food security, and a Principal Component Analysis (PCA) to select key indicator variables from logically related sets of variables to avoid collinearity.

## Results

### Regressions of self‐assessed resilience and food security

We set out to examine the results from the regressions on self‐assessed resilience and food security. We consider how self‐assessed resilience is influenced by specific variables to explore correlations with variables such as climate shocks experienced, access to weather forecasts, resource conflicts reported and degree of involvement in development activities. We also used these variables to estimate correlates of the food security measure to compare with the resilience regression results (see Tables 1 and 2).

**Table 1:** Parameter estimates from the regressions of predictor variables on self‐assessed resilience as responses in Mali and Senegal

Variables	Mali			Senegal		
	Coefficients		SE	Coefficients		SE
**Intercept**						
Log village population	0.05	ns	1.78	1.03	***	0.37
River‐based ecosystem	−0.02	ns	0.25			
North—Ferlo				0.03	ns	0.77
Middle—peanut basin				0.30	ns	0.75
Household head sex	1.45	ns	0.98	0.96	*	0.55
Household size	−0.03	ns	0.06	0.09	ns	0.07
Number of dependants	0.08	ns	0.06	0.01	ns	0.12
Agriculture as primary income	1.84	***	0.40	−1.23	ns	1.01
Livestock as primary income	0.60	ns	0.40	−1.99	ns	1.64
Commerce as primary income	1.33	ns	1.02	−0.22	ns	0.94
Income diversification	0.28	**	0.12	0.83	***	0.23
Number of shocks	−0.43	**	0.17	−0.51	**	0.21
Access to cultivation zones	1.77	***	0.57	1.12	*	0.68
Access to communal resources	−1.50	ns	1.50	−0.28	ns	0.44
Access to weather forecast	0.44	ns	0.44	1.29	***	0.40
Access to agriculture water points	−0.04	ns	0.04	−0.72	ns	0.69
Access to animal water points	1.54	***	0.31	0.40	ns	0.53
Access to markets	0.02	ns	0.13	−0.03	ns	0.12
Access to market information	0.45	***	0.11	−0.29	ns	0.19
Access to livestock inputs	0.15	ns	0.18	0.12	ns	0.12
Access to financial services	0.63	ns	0.47	0.42	ns	0.53
Number of community facilities available	−0.19	**	0.09	0.04	ns	0.15
Number of natural resources available	0.13	ns	0.08	0.12	ns	0.27
Number of conflicts over natural resources	0.05	ns	0.12	0.53	*	0.27
Average involvement in development activities	0.66	***	0.08	0.05	ns	0.25
Constant cut1	5.88	ns	5.39	8.26	*	4.34
Constant cut2	9.15	*	5.54	10.62	**	4.30
Constant cut3	11.47	**	5.54	12.84	***	4.84

**Notes**: Robust standard errors are clustered at the village level. SE = standard error. Significance values: ‘ns’ = non‐significant; ‘*’=P < 0.05; ‘**’=P < 0.01; ‘***’=P < 0.001.

**Source:** authors.

**Table 2:** Parameter estimates from the regressions of predictor variables on food security as responses in Mali and Senegal

Variables	Mali			Senegal		
	Coefficients		SE	Coefficients		SE
**Intercept**						
Log village population	0.01	ns	0.05	0.07	ns	0.57
River‐based ecosystem	−0.09	ns	0.06			
North—Ferlo				−0.08	ns	0.07
Middle—peanut basin				0.09	ns	0.08
Household head sex	0.01	ns	0.16	0.05	ns	0.07
Household size	0.02	**	0.01	0.01	ns	0.01
Number of dependants	0.01	ns	0.01	−0.01	ns	0.01
Agriculture as primary income	0.21	***	0.06	−0.18	***	0.06
Livestock as primary income	−0.08	ns	0.07	−0.10	ns	0.06
Commerce as primary income	0.12	ns	0.17	−0.04	ns	0.05
Income diversification	0.07	***	0.02	0.08	***	0.03
Number of shocks	−0.13	***	0.04	0.00	ns	0.00
Access to cultivation zones	0.21	*	0.12	−0.04	ns	0.04
Access to communal resources	−0.42	**	0.19	−0.12	**	0.05
Access to weather forecast	0.07	ns	0.06	0.12	***	0.04
Access to agriculture water points	−0.02	ns	0.08	−0.13	**	0.05
Access to animal water points	−0.03	ns	0.03	0.04	ns	0.05
Access to markets	0.02	ns	0.02	0.00	ns	0.02
Access to market information	0.12	***	0.03	−0.03	*	0.02
Access to livestock inputs	0.03	ns	0.04	0.03	**	0.01
Access to financial services	−0.17	ns	0.12	0.07	ns	0.04
Number of community facilities available	0.04	***	0.01	−0.02	*	0.01
Number of natural resources available	0.00	ns	0.02	0.02	ns	0.03
Number of conflicts over natural resources	0.02	ns	0.03	0.06	**	0.03
Average involvement in development activities	0.04	**	0.02	0.02	ns	0.02
Constant	1.20	**	0.58	1.63	***	0.47

**Notes**: Robust standard errors are clustered at the village level. SE = standard error. Significance values: ‘ns’ = non‐significant; ‘*’=P < 0.05; ‘**’=P < 0.01; ‘***’=P < 0.001.

**Source:** authors.

In Mali and Senegal, self‐assessed resilience is largely associated with household socio‐demographic characteristics. While village size is positively linked with resilience in Senegal, agro‐ecological zones do not come up as a significant factor in either country. In both countries, income diversification, number of shocks experienced and access to cultivation zones are significant correlators. However, other correlators differ. Agriculture as primary income, access to animal water points, access to market price information and household's level in community involvement correlate positively with resilience. In contrast, there is a negative relationship between the number of community infrastructure facilities available, such as grain storage, rainwater infrastructure and perceptions of resilience in Mali. In Senegal specifically, households headed by men are more likely to feel strongly resilient to climate change and adverse shocks. Additionally, households with access to weather forecast information reported higher levels of resilience. Surprisingly, households experiencing more conflict over natural resources were more likely to report feeling more resilient.

We found a higher number of significant predictor variables for food security than for self‐assessed resilience. There are some overlaps in predictors between countries yet several differences are highlighted. Attributes of food security in Mali align strongly with seven of the eight statistically significant attributes of self‐assessed resilience. This includes the significance and direction of correlations between response variables and agriculture as their main source of income, income diversification, number of shocks experienced, access to cultivation zones, access to market information, number of community facilities available and involvement in development activities. In addition, household size positively correlates with food security. However, access to communal resources, such as grazing fields and fishing ponds, is also negatively associated with months of food security. This unexpected pattern also emerges in Senegal. In both countries and for both regressions, income diversification is positively correlated with the dependent variable.

A number of differences in significance and direction of correlation appear between the two countries—namely, some variables positively correlated with food security in Mali are negative in Senegal: agriculture as primary income, access to market information and access to community facilities. Additionally, we found access to weather forecasts, access to livestock inputs and number of conflicts experienced to be positive predictors of resilience in Senegal but not in Mali. In Senegal, households with access to water points for agriculture had fewer months of food security. As such, attributes of self‐assessed resilience and food security in Senegal do not align as well as they did in Mali, with the only common predictors being income diversification, access to weather forecasts and number of conflicts over resources experienced.

## Discussion

Most resilience frameworks state that place specificity and context are critical in defining resilience, but it is not clear how this principle translates into practical assessments that are robust, that inform understanding of local changes and improve programming (downwards accountability) and that respond to donor priorities such as aggregation (upwards accountability). This study was a first step in exploring how local perceptions of resilience in Mali and Senegal vary according to household‐ and village‐level demographic, social and economic variables. We find that, while both response variables are for the most part aligned in Mali, this is much less the case in Senegal. Additionally, we find correlates for the respective dependent variables between countries to exhibit different patterns, pointing to different underlying effect mechanisms. The differing patterns in the results comparing self‐assessed resilience and food security, as relating to well‐being, illustrate that the interplay between domains or attributes of resilience and well‐being are still unclear. Unlike normative assumptions often held in development discourse, results highlight that directional linkages are not straightforward.

These results provide us with the opportunity to develop further our understanding in the communities where we work as we continue our research. The analysis of longitudinal data and contribution to changes in the responses over time will allow a deeper understanding of such linkages and of trajectories for more resilient pathways of development. The regressions unpack dynamics of variation within self‐assessed resilience groups and help us understand the profile of households that reported greater resilience and food security.

### Resilience and its correlates

In Mali and Senegal, the main findings are that households with a diversity of other livelihoods and with access to cultivation zones felt more resilient. In Mali, those that had agriculture as their primary income also felt more resilient, whereas in Senegal male household heads felt more resilient. This is a generalisable result as wives of the same households reported lower resilience than their husbands did (McPeak and Little, 2017). We interpret that agricultural households that are sedentary are better off than pastoralist and mobile households, with more diverse livelihoods (a well‐known strategy in the Sahel) enabling better risk management. Self‐assessed resilience decreased for households that had experienced a higher number of shocks, which is intuitive.

However, in contrast with what was found in Mali, households experiencing more conflicts over natural resources in Senegal reported higher resilience, along with higher food security. Further disaggregation of the data showed that many of these observations are driven by a few villages where fishing, herding and farming are all represented in the sample and, with greater heterogeneity in resources and livelihood activities, there is both greater livelihood diversification (a key resilience strategy) *and* more to contest. The sites where multiple resources are present are generally among the project's most western sites, situated closest to the city of Kaolack. There may, therefore, also be a conflict driver arising from resource pressure associated with the increasing outward sprawl of this city—this being consistent with the finding that higher resilience is reported in larger villages. Thus, proximity to Kaolack (and larger villages in general) may enable a greater diversity of livelihood strategies and access to markets that support greater food security and resilience, along with more resource conflicts.

Similarities in three of the significant variables between Mali and Senegal point to shared dynamics across the landscape. Yet the respectively remaining four and three other significant variables highlight the presence of contextual nuances. The scale at which these nuances interact is difficult to assess based on one year of data, yet larger economic and security trends can explain some patterns. For example, the lack of significance of household involvement in development activities denotes that there are fewer interventions operating in Kaffrine than there are in Mopti. Such trends can play a role in influencing different dynamics of resilience and food security linked to livelihood strategies, pointing to the importance of contextual subjectivities in measuring resilience.

### Food security and its correlates

Results are very similar when comparing correlates of self‐assessed resilience and food security in Mali, unlike in Senegal, where only three predictors are shared between response variables. Again, when comparing countries, we observe both similarities and differences, with five common predictors across the study sites out of nine for each country. In Mali, we observe differences only in household size, where larger households have better food security instead of less; in access to animal water points, where access is correlated with fewer months of food security; and in number of facilities available, where households in villages with more facilities report better food security.

In Senegal, some factors significant for self‐assessed resilience are absent as correlates for food security: village population size, sex of household head, number of shocks experienced and access to cultivation zones. Other factors are significantly only correlated to food security and not self‐assessed resilience, such as access to weather forecast information and the number of conflicts over resources being associated with increased food security. This is also the case for agriculture as primary income, suggesting that patterns differ between the two countries in linkages between main livelihood strategies, resilience and food security.

Surprisingly, access to communal resources is correlated in both countries with lower food security, while access to cultivation zones is correlated with higher resilience and food security in Mali. Such different correlations between access to cultivated areas and access to communal resources show the importance of management systems for natural resources in securing resilience, despite their locations across agro‐ecological zones.

Communal resources include grazing fields, fishing regions and non‐timber forest products, while cultivation zones include irrigated vegetable and rice plots, and land rehabilitated after water conservation measures. The notable difference that the latter is not only irrigated but also likely managed by a socio‐professional group in the village similar to a club good, whereas communal resources are more similar to open access. In this sense, households that have access to communal resources (under an open‐access framework) felt more vulnerable than those that rely on cultivation zone resources (where access may be limited to members of a socio‐professional group and investments are made to improve resources). Thus there may be an implication that access to communal resources is considered to be associated with lower resilience, in that someone who does not have to rely on community resources, owing to sufficient private resources, perceives themselves to be more resilient. This is a line of research that merits further focus.

The patterns of resilience and food security shown here are in line with other studies documenting social changes in the Sahel (Elmqvist and Olsson, 2007; McPeak and Little, 2017). Thus, factors linked to resilience are generally linked to positive social outcomes from other types of development interventions, whether climate‐focused or not. The findings also show that resilience and food security are driven by access to and management of resources at the local level, yet not necessarily affected by agro‐ecological landscape resource availability. With the exception of a few variables (income diversification in four of the four regressions and number of shocks in three of the four regressions), we find that many of the variables that would be predicted to correlate strongly with self‐assessed resilience, and thus indirectly with food security, matter in some cases but not in others.

### Implications for using the subjective measure

There are a few surprises, where results we expected to be significant and of a given sign either are not statistically significant or signed contrary to our expectations. For example, agro‐ecological zones were not significant predictors of resilience, and rather pointed to the importance of household‐level factors influencing resilience and food security. While resilience is framed in the context of climate shocks, local communities cannot easily separate their experience of different shocks and stresses (McPeak et al, 2017). Yet failure to consider the multiple drivers of vulnerability can increase the risk of maladaptation or unsuccessful adaptation (Adger et al., 2011). As such subjective measures must be based on in‐depth participatory qualitative assessments of local situations and paired with additional factors in order to derive the underlying mechanisms related to climate per se. This study underlines the multidimensionality and context‐centred nature of resilience; no single indicator can significantly capture the geographic nuances within which resilience operates, either for use as a local evaluative tool or for wider aggregation in global frameworks.

Although the focus is on the individual and the household, resilience at these levels also depends on the resilience of the community, systems and ecosystems in which they live. Social context and cultural settings are part of individuals’ conceptualisations of resilience, reinforcing the need to address subjectivity in resilience versus only observable factors (Jones and Tanner, 2017). Using perceptions of resilience, which have been grounded in participatory assessments, to monitor and evaluate interventions as part of a set of other observable factors can help ground projects in the local circumstances and avoid top‐down decision‐making, which runs counter to the contextualised nature of resilience—as shown by differences observed between Mali and Senegal.

By comparing a perception‐based resilience indicator and a locally understood well‐being indicator (food security), this study shows that using either measure in isolation, for example as a local indicator within government M&E systems, is unlikely to capture the complexity needed to reflect adaptation processes and resilience outcomes. For local purposes, a small set of indicators, including self‐assessed perceptions, food security and a small number of observable variables tailored to the livelihood group or intervention in question, may meet the standards of evidence needed while also being feasible in local M&E contexts.

### Implications for using the subjective measure

In conclusion, this study raises two main questions linked to the design of frameworks measuring resilience and well‐being. First, it questions the validity of normative linkages leading from resilient outcomes to well‐being impacts. While this study has not unearthed temporal trends, the lack of patterns between the two indicators underlines the complex nature of development trajectories. Both well‐being and resilience are multidimensional concepts that must be understood through interacting and fluctuating factors with often shifting baselines. Assuming such linkages can put an emphasis on net impacts instead of creating positive mechanisms of change, in which resilient processes are an end in themselves.

Second, the study questions how far the objective of aggregation should shape the design of local resilience indicators, and its validity and usefulness given that aggregated indicators lose the nuanced contextualisation within which they are being measured. As such, the aggregated results of either resilience or food security across countries could lead to maladaption if not disaggregated down to village level to explain particular unintuitive social mechanisms (such as resilience and conflicts experienced). The BRACED KPI4 framing is theoretically aggregable, but the numbers being aggregated are made up of different indicators compiled and weighted in different ways. It is unclear therefore exactly how this information can be used beyond a high‐level figure for reporting, and how reflective it is of actual shifts in resilience on the ground. There is also increasing debate at the international level about aggregating programme results and national progress towards targets such as the adaptation goal (Craft and Fisher, 2018). The differences we see in the results in the self‐perceived measures, and in other correlates of well‐being within and between contexts, suggest the need to exercise extreme caution in aggregating indicators across contexts under attempts to account for progress toward adaptation at programmatic and national levels.

## Acknowledgements

The authors wish to acknowledge contributions from all members of the NEF/IIED/IED Afrique consortium of the DCF BRACED project.

## Endnotes

2 Representing local producers, elected communal officials and agents from the technical services for agriculture, livestock, fisheries, water and forestry, etc.

3 See detailed reports.
